# Mitogenomic analysis and phylogenetic relationships of Agrilinae: Insights into the evolutionary patterns of a diverse buprestid subfamily

**DOI:** 10.1371/journal.pone.0291820

**Published:** 2023-09-28

**Authors:** Xuyan Huang, Zhonghua Wei, Jiawei Lu, Aimin Shi

**Affiliations:** 1 College of Life Sciences, China West Normal University, Nanchong, Sichuan, China; 2 Mental Health Center of Nanchong, Nanchong, Sichuan, China; ICAR Research Complex for Eastern Region, INDIA

## Abstract

Agrilinae is the largest subfamily in Buprestidae, which includes the four tribes, namely Coraebini, Agrilini, Aphanisticini, and Tracheini. However, there is a need to verify the evolutionary relationships among the taxa in Buprestidae. Thus, to explore the phylogenetic position of Aphanisticini, the mitochondrial genomes of *Endelus continentalis* and *Cantonius szechuanensis* were sequenced using next-generation sequencing technology. Three other mitogenomes of agriline beetles, *Agrilus discalis*, *Sambus kanssuensis*, and *Habroloma* sp., were also sequenced for the phylogenetic analyses. The divergence time of Buprestidae was estimated based on the mitogenomes. The general features of the known mitogenomes of Agrilinae were compared, analyzed, and summarized. Out of these five species, *S*. *kanssuensis* had the shortest mitogenome length (15,411), while *Habroloma* sp. had the longest (16,273). The gene arrangement of the five new sequences was identical to that of the reported buprestid mitogenomes. The Ka/Ks ratios of *Meliboeus* (0.79) and *Endelus* (0.78) were significantly larger than those of the other agriline genera. The results of the phylogeny indicated that Aphanisticini was more closely related to Tracheini and that the genus *Sambus* separated from the base of the Agrilinae clade at about 130 Ma. Moreover, Aphanisticini and Tracheini diverged at around 26 Ma.

## Introduction

Agrilinae is the largest subfamily in the family Buprestidae and even in the class Insecta, which includes four tribes, namely Coraebini Bedel, 1921, Agrilini Laporte, 1835, Aphanisticini Jacquelin du Val, 1859, and Tracheini Laporte, 1835, in the modern classification systems [1]. In the subfamily Agrilinae, most of the adults feed on the fresh leaves of woody and herbaceous plants [2], and a few species also feed on pollen (for example, some species of the genus *Meliboeus*). However, the larvae of the tribes Agrilini and Coraebini are wood-boring [3–6], and the larvae of the tribes Aphanisticini and Tracheini are leaf miners [7–11].

The phylogenetic analyses of Buprestidae based on morphological and molecular data were summarized in previous studies [12–14]. Although there have been significant contributions to the classification systems, the buprestid phylogeny is still not well resolved. In addition, there has been little molecular phylogenetic research on the higher taxa. In 2015, Evans et al. provided a large-scale molecular phylogeny of Buprestidae based on two nuclear and two mitochondrial genes, suggesting that the species of the tribe Coraebini were dispersed throughout Agrilinae, with a strong support value, and the independent origins of leaf-mining within the subfamilies Polycestinae and Agrilinae [15]. However, the genera *Sambus*, *Endelus*, and *Cantonius* were not included in that study. Moreover, the phylogenetic position of the genus *Sambus* remains controversial based on the classification system [6,16] and molecular phylogenetic analyses of Huang et al. [12] and Wei et al. [14].

Consequently, to estimate and verify the evolutionary relationship of the higher taxa in Agrilinae, the mitogenomes of five Agrilinae species, namely *Agrilus discalis* Saunders, 1873, *Endelus continentalis* Obenberger, 1944, *Cantonius szechuanensis* Obenberger, 1958, *Sambus kanssuensis* Ganglbauer, 1889, and *Habroloma* sp., were sequenced using next-generation sequencing. The general features and sequence heterogeneity of the Agrilinae mitogenomes were provided. The sequence heterogeneity of all the known buprestid mitogenomes was also analyzed.

## Materials and methods

### Samples and sequencing

The specimens of *Agrilus discalis* and *Endelus continentalis* were collected from the Dayaoshan Mountains in Guangxi Zhuang Autonomous Region, China, on 23 April 2021 and 20 April 2021, respectively. The *Cantonius szechuanensis* specimen was collected from the Jigongshan Mountains, Mabian County, Sichuan Province, on 10 June 2022. Additionally, the specimens of *Sambus kanssuensis* and *Habroloma* sp. were collected from Yintiaoling, Wuxi County, Chongqing, on 29 June 2022. These specimens were preserved in 95% alcohol at -24°C in the specimen collection at China West Normal University, Nanchong, China. Then, next-generation sequencing was performed by Beijing Aoweisen Gene Technology Co. Ltd. (Beijing, China) to obtain the raw data.

### Sequence assembly, annotation, and analysis

To obtain the target reads from the raw data, the adapter sequence and low-quality bases were removed using Trimmomatic v. 0.36 [17]. Then, the target reads were assembled using IDBA-UD v. 1.1.1 and Celera Assembler v. 8.3 [18]. The identification of the open reading frames led to the identification of the protein-coding genes (PCGs). The secondary structures and locations of the transfer RNA (tRNA) genes were identified using tRNAScan-SE server v. 1.21 [19] and MITOS WebServer [20]. By examining the boundaries of the tRNA genes, the ribosomal RNA genes (rRNAs) and control region were identified. The de novo assembly of the mitochondrial contigs was conducted using Geneious v. 11.0.2 [21]. The nucleotide composition of the mitogenomes and the codon usage of the PCGs were analyzed using MEGA v. 12.0.0 [22]. Next, the strand asymmetry of the mitogenomic sequences was calculated using the formula that was provided by Perna and Kocher [23]. The values of the nonsynonymous substitution (Ka), synonymous substitution (Ks), and the ratio of Ka/Ks of the PCGs in the agriline genera were calculated using *Ptosima chinensis* Marseul, 1867 as the reference sequence. The sequence heterogeneity of the nucleotide matrix (PCG and PCGRNA datasets) was also calculated and analyzed using AliGROOVE v. 1.06 [24]. These five newly generated mitogenomes are available on Genbank (Genbank accession no. OQ784264, OQ784265, OQ784266, ON644870, and OL702762; SRA accession no. SRR25120363, SRR25107774, SRR25117213, SRR25083110, and SRR25083222).

### Phylogenetic analysis and divergence time estimation

In this study, a total of 28 buprestid species were used in the phylogenetic analyses (S1 Table), with two outgroups (*Heterocerus parallelus* Gebler, 1830 and *Dryops ernesti* Gozis, 1886). The sequences of the PCGs and RNAs were aligned and trimmed using ClustalW [25] and trimAl v. 1.2 26 [26], respectively. The ambiguous positions in the aligned sequences were removed using Gblocks v. 0.91b [27]. Then, the sequences of the same species were concatenated using the ‘Concatenate Sequence’ tool in PhyloSuite v. 1.2.2 [28]. The sequence matrixes (13 PCGs and two RNAs) were used for the phylogenetic analyses. The phylogenetic trees were reconstructed using IQ-tree v. 1.6.8 [29] and MrBayes v. 3.2.6 [30] based on the Maximum-likelihood (ML) and Bayesian inference (BI) methods, respectively. The parameters to construct the phylogenetic trees were as follows, ML: bootstrap: ultrafast, number of bootstraps: 5000, maximum iterations: 1000, minimum correlation coefficient: 0.90, replicates: 1000; BI: generations: 2,000,000, sampling frequency: 100, number of runs: 2, number of chains: 4, and burnin fraction: 0.25. The phylogenetic trees were visualized and edited using Figtree v. 1.4.3. In order to reduce the attraction of long branches, we constructed another BI tree based on cd12 + rRNAs using the CAT-GTR model in PhyloBayes 4.1 [31].

The divergence time of Buprestidae was estimated using Beast v. 2.6.6 [32] based on the sequence matrix (PCGRNA dataset). The fossil taxa *Sinoparathyrea bimaculata* Pan, Chang & Ren, 2011 and *Agrilus corrugatus* Waterhouse, 1889 were used to calibrate the node age [33,34]. The tracer v. 1.7.1 software was used to test the convergence, with 10% burn-in. The TreeAnnotaor v. 2.6.0 software was used to generate the maximum clade credibility tree, with a burn-in of 10% and a posterior probability limit of 50.

## Results

### General features of the agriline mitogenomes

**Fig 1:**
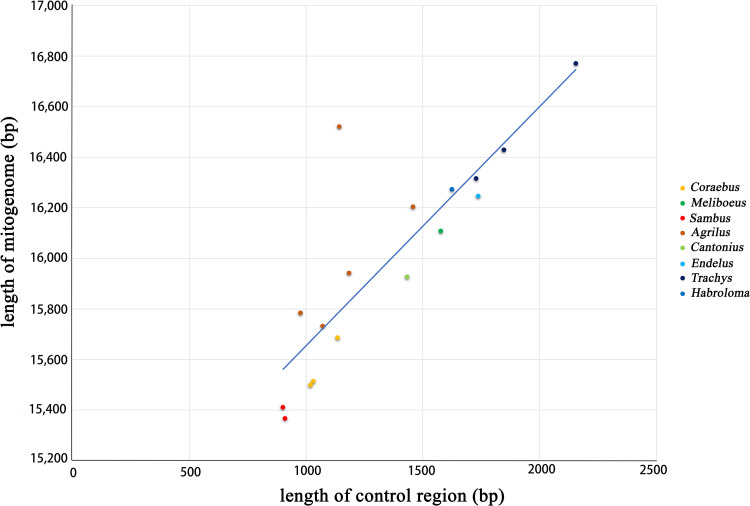
The length of the complete mitogenomes and control regions of 17 Agrilinae species.

The complete mitogenomes of the representative genera of the four tribes (Coraebini, Agrilini, Aphanisticini, and Tracheini) were all analyzed in this study (S1 Table). The basic composition of the five newly generated sequences is presented in Table 1. Among the 17 Agrilinae species, all had complete mitogenomes. These mitogenomes all had 37 typical genes (13 PCGs, 22 tRNAs, and two rRNAs) and a control region (A + T-rich region). The length of the 17 complete mitogenomes ranged from 15,411 bp (*Sambus kanssuensis*) to 16,771 bp (*Trachys variolaris* Saunders, 1873), with an average length of 16,000 bp. The variation in the mitogenomic length was mostly caused by various length variations in the control region (Fig 1). The phenomenon of gene rearrangement was not supported in these 17 Agrilinae species.

**Table 1:** General features of the five Agrilinae mitogenomes that were provided in this study.

Species	*A*. *discalis*	*E*. *continentalis*	*C*. *szechuanensis*	*S*. *kanssuensis*	*Habroloma* sp.
Whole length (bp)	15,784	16,246	15,927	15,411	16,273
Whole (A + T) %	74.59	75.60	73.09	72.40	73.99
AT-skew	0.11	0.13	0.11	0.10	0.11
GC-skew	-0.20	-0.18	-0.18	-0.17	-0.20
PCGs length (bp)	11,161	11,078	11,096	11,089	11,145
PCGs (A + T) %	73.19	74.29	70.44	70.79	72.78
tRNAs length (bp)	1467	1358	1438	1436	1453
tRNAs (A + T) %	75.19	76.36	74.48	74.09	74.19
rRNAs length (bp)	1977	1967	2007	1984	2000
rRNAs (A + T) %	78.10	81.24	78.77	76.81	77.80
Control region length (bp)	976	1737	1433	901	1625
Control region (A + T) %	81.05	76.68	84.44	79.91	76.92
Gene overlap region	11	9	10	12	10
Gene overlap range (bp)	1–16	1–7	1–7	1–8	1–8
Gene overlap size (bp)	37	21	31	37	33
Intergenic region	8	7	7	10	10
Intergenic range (bp)	1–18	1–55	1–18	1–17	1–27
Intergenic size (bp)	44	71	32	35	78

All 17 mitogenomes of Agrilinae had a high AT nucleotide bias with the A + T content ranging from 68.42% (*Coraebus diminutus* Gebhardt, 1928) to 75.60% (*Endelus continentalis*). Additionally, these mitogenomes had a positive AT skew (Table 1) ranging from 0.08 (*Agrilus mali* Matsumura, 1924) to 0.13 (*Endelus continentalis*) and a negative GC skew.

**Fig 2:**
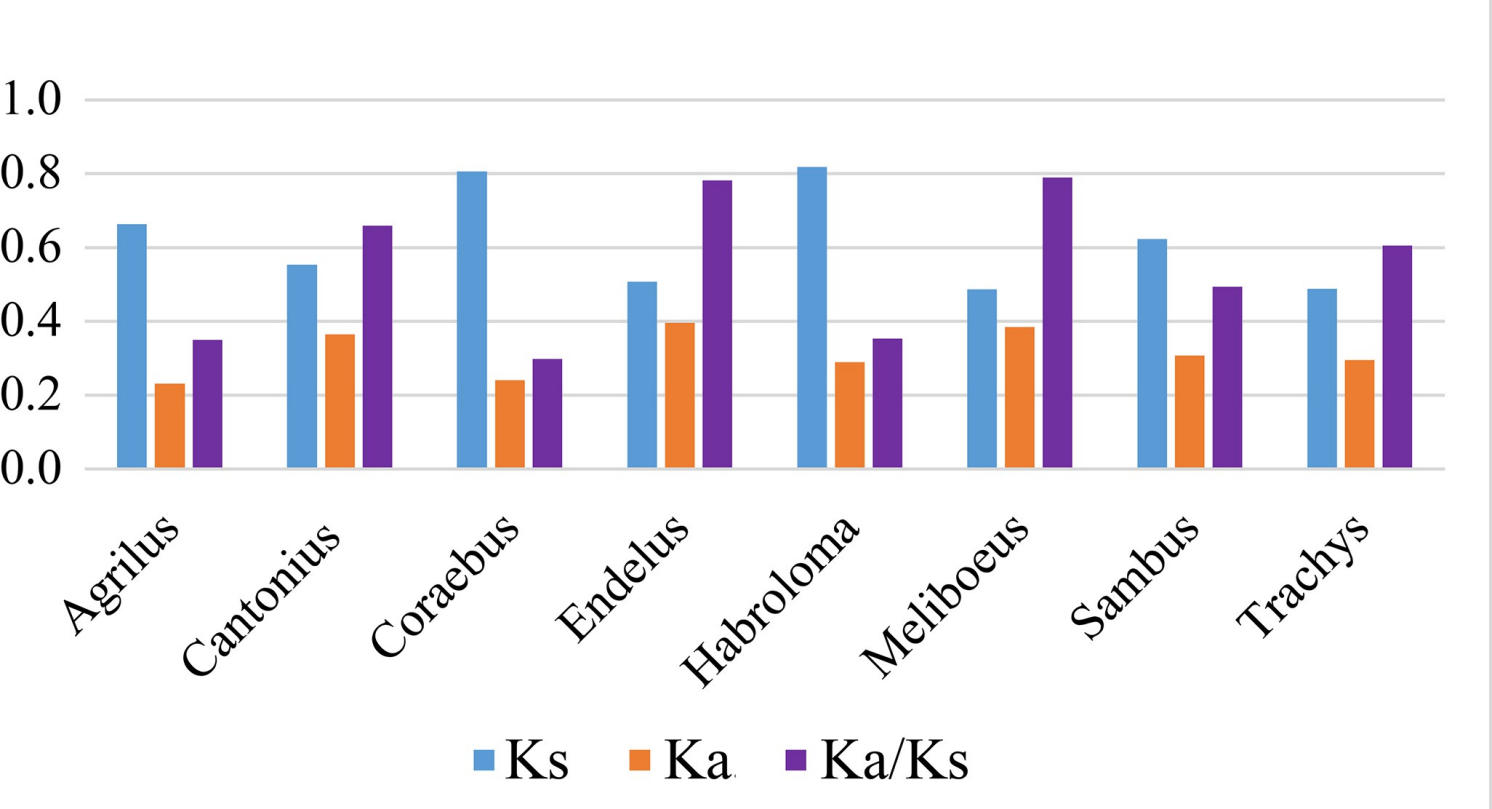
The Ka, Ks and Ka/Ks of the eight genera of Agrilinae.

In Agrilinae, most of the PCGs started with a typical ATN codon (ATA, ATT, or ATG), while a small number of the start codons were TTG (*NAD1*) and GTG (*NAD4L* and *NAD5*). The stop codons had three types, TAA, TAG, and a single T, of which the incomplete stop codon T—was completed by the addition of 3’ A residues to the mRNA. The values of Ka, Ks, and Ka/Ks of the PCGs in the agriline genera are presented in Fig 2 using *Ptosima chinensis* as the reference sequence. The Ka/Ks of *Meliboeus* (0.79) and *Endelus* (0.78) were distinctly higher than that of the other agriline genera.

In the 17 Agrilinae species, the length of the individual genes ranged from 58 (*trnS2*) to 72 bp (*trnW*). The dihydrouridine arm of *trnS1* was observed as a simple loop even though all other tRNAs were able to fold into the typical cloverleaf secondary structure. The length of *16S* ranged from 1250 bp (*Trachys auricollis* Saunders, 1873) to 1365 bp (*Agrilus planipennis* Fairmaire, 1888), while the length of *12S* ranged from 696 bp (*Sambus femoralis* Kerremans, 1892) to 803 bp (*Coraebus cavifrons* Descarpentries & Villiers, 1967). The size of the control region ranged from 901 (*Sambus kanssuensis*) to 2155 bp (*Trachys variolaris*) among the 17 Agrilinae mitogenomes.

In these five new sequences, the largest intergenic regions ranged from 17 bp (*Sambus kanssuensis*) to 55 bp (*Endelus continentalis*) and were located between *trnS2* and *NAD1*. Moreover, the gene overlap size ranged from 21 bp (*Endelus continentalis*) to 37 bp (*Agrilus discalis*).

### Sequence heterogeneity, phylogenetic relationships, and divergence time

**Fig 3:**
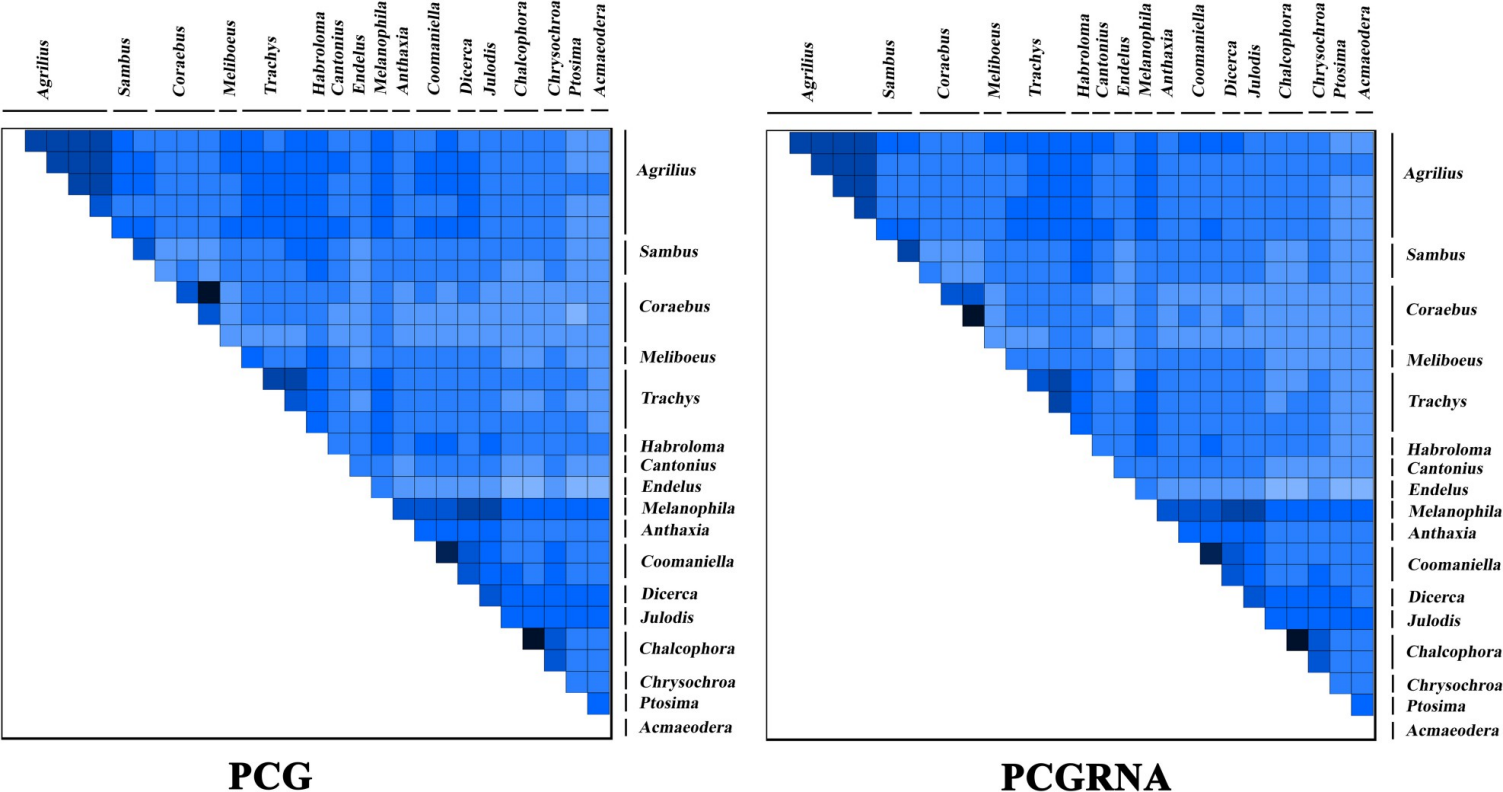
The heterogeneity of the two datasets (PCGs and PCGRNA) for Buprestidae.

The degree of heterogeneity of the PCG and PCGRNA datasets is presented in Fig 3. The results indicated that the sequence heterogeneity of the PCGs was higher than that of the PCGRNA. The sequence divergence of *Coraebus* and *Endelus* was higher than that of the other buprestid genera.

**Fig 4:**
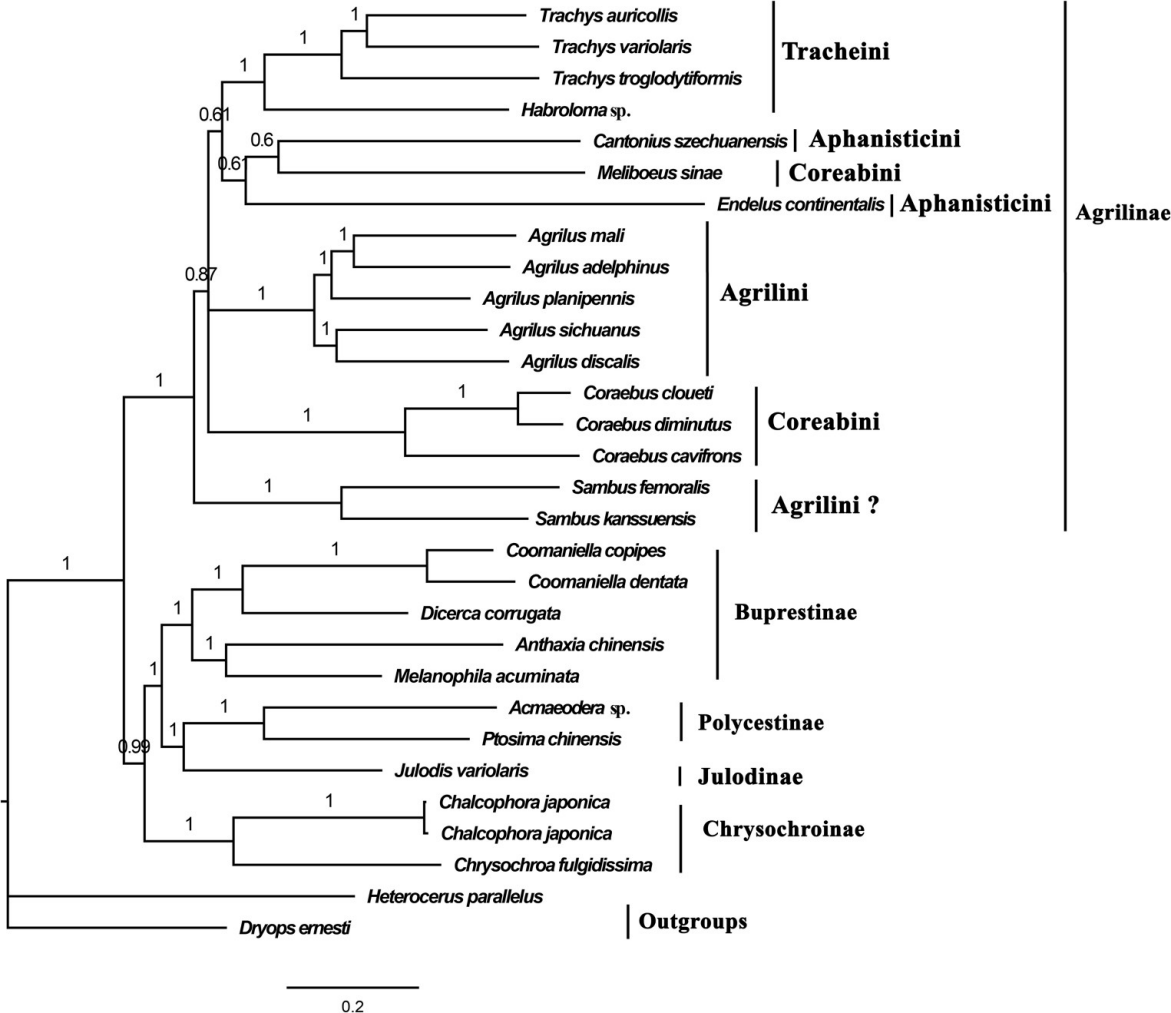
The Bayesian inference (BI) tree of the studied species of Buprestidae based on the PCGRNA dataset. The values above the nodes represent the Bayesian posterior probabilities.

The topology of the BI and ML trees was identical except for Agrilinae (Figs 4, S6 and S7). All the buprestid taxa clustered together and formed a clade, with high bootstrap values (BI: 1; ML: 100). The five subfamilies of Buprestidae formed two sister groups: (Agrilinae) and (Chrysochroinae + ((Julodinae + Polycestinae) + Bupretinae)), with high support values (BI: 0.99, 1; ML: 43, 100). In Agrilinae, *Sambus* was separated at the base of the agriline clade. Coraebini was a paraphyletic group because *Meliboeus* clustered with *Cantonius* (Aphanisticini). When compared with Coraebini and Agrilini, Aphanisticini was more closely related to Tracheini. The BI tree (S7 Fig) based on the CAT-GTR model showed that Aphanisticini is paraphyletic: the *Endelus* is close to *Agrilus* and the *Cantonius* is close to *Meliboeus*.

**Fig 5:**
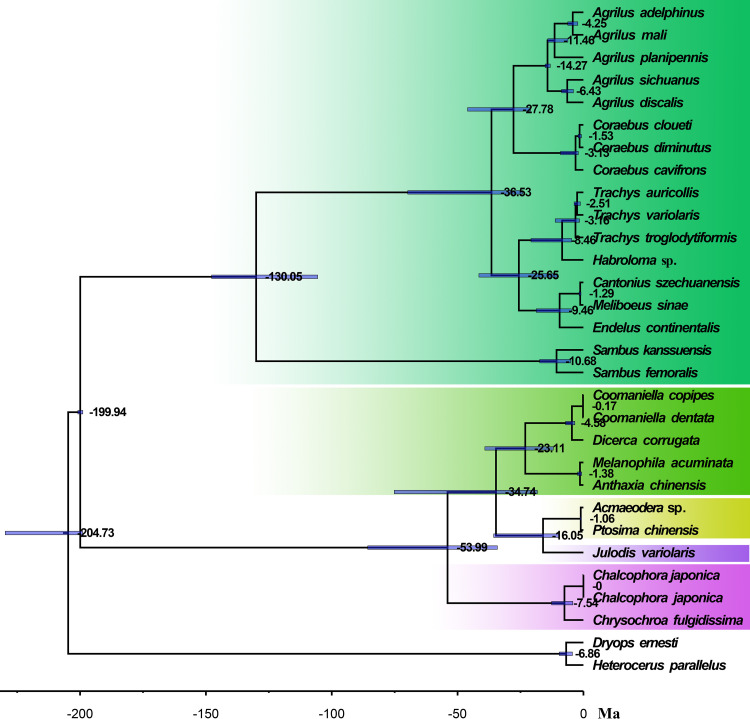
The dated phylogenetic tree of Buprestidae based on the PCGRNA dataset. A time scale is provided, and Ma refers to one million years.

Based on the results of the divergence time (Fig 5), Agrilinae was separated from the other buprestid families at about 200 Ma, and *Sambus* were separated from the other agriline genera at around 130 Ma. The leaf miners Tracheini and Aphanisticini were divided at about 26 Ma, while the wood-boring Agrilini and Coreabini were divided at around 28 Ma.

## Discussion

### Characteristics of the mitogenomes in Agrilinae

The subfamily Agrilinae includes four tribes, namely Coraebini, Agrilini, Aphanisticini, and Tracheini, which are distributed worldwide [1]. There are 17 complete mitogenomes that have been reported in this subfamily, including five new mitogenomic sequences that were provided in this study. Among them, the mitogenomes of the tribe Aphanisticini (*Cantonius szechuanensis* and *Endelus continentalis*) and the genus *Habroloma* were the first reported for these taxa. These five new sequences had 37 typical genes and a control region, which is consistent with those of most insects that were reported previously [35,36]. No gene rearrangement occurred in these mitogenomes, which supports the results of Wei et al. [14]. The length of the complete mitogenomes was mostly attributed to the length of the control region, which is consistent with a previous study [37]. All the Agrilinae mitogenomes had positive AT skew and negative GC skew, which is consistent with those of most reported insects [37–44]. In Agrilinae, most of the PCGs had normal start and stop codons, while a few genes had unconventional codons, such as: *NAD1* [13,14] and *COII* [45] that were initiated with GGT and *NAD4L* and *NAD5* that started with GTG [13].

The ratio of Ka to Ks was used to estimate the evolutionary rate, which was proposed by Hurst [46]. All the Ka/Ks ratios of the genera in Agrilinae were lower than 1, suggesting that these genera are under purifying selection. Although *Agrilus* had the largest number of species, the Ka/Ks ratio of this genus was lower than that of other agriline genera, except for *Coraebus*.

### Tribe-level phylogenetic relationships

Although a large-scale molecular phylogenetic analysis of Buprestidae was conducted by Evans et al. [15], the evolutionary relationships of the higher taxa in Agrilinae had yet to be addressed because of the taxa of Coraebini that were dispersed throughout Agrilinae. In this study, the evolutionary relationship of the five subfamilies in Buprestidae was (Agrilinae + (Chrysochroinae + ((Julodinae + Polycestinae) + Bupretinae))), which is consistent with a previous study [14]. All the species of Agrilinae formed an independent clade with high support values, and the taxa of *Sambus* formed a single clade at the base of the Agrilinae clade, which supports the previously suggested topology of Kubáň [16], who regarded *Sambus*, *Parasambus* and *Pseudagrilus* as incertae sedis genera. To completely address this problem, the molecular data of the genera *Parasambus* and *Pseudagrilus* are needed. The results also suggest that *Meliboeus* (Coraebini) is more closely related to *Cantonius* (Aphanisticini) than *Coraebus* (Coraebini). Additionally, some previous studies have confirmed that the heterogeneity of mitogenomic sequences in certain groups may lead to the erroneous grouping of unrelated taxa [47–50], so the phylogenetic positions of *Meliboeus* may be misplaced. Mito-nuclear discordance is ubiquitous in many study systems, certainly in the presence of incomplete lineage sorting or introgression [50–53].

Based on the estimated divergence time of Buprestidae, the larval feeding habit of its ancestry is wood-boring and some groups have shifted to leaf mining in Agrilinae. Moreover, the origins of leaf mining are independent within Polycestinae and Agrilinae [15].

Overall, further research on the evolutionary relationships of Buprestidae is needed to confirm the relationships based on more mitogenomic and nucleotide data.

## Conclusions

In this study, the mitogenome sequences of the five newly sequenced Buprestidae species (15,411–16,273 bp) were provided, and their mitogenome were compared and analyzed. Among them, *Cantonius szechuanensis* and *Endelus contantalis* were the first complete mitogenome sequences that have been reported in the tribe Apaniticini. In addition, these five sequences have a positive AT-skew.

Among all tRNAs, only *trnS1* has no typical clover-leaf structure, and its dihydrouridine arm is observed to be a simple ring. The rearrangement phenomenon has not been detected in this study. The results indicated that the sequence heterogeneity of the PCGs was higher than that of the PCGRNA, and the degree of sequence divergence for *Coraebus* and *Endelus* was significantly higher than that of the other buprestid genera. However, the Ka/Ks ratio of *Meliboeus* was the largest, while that of *Coraebus* was the smallest. The phylogenetic results showed that Aphanisticini is more closely related to Trainini, and *Meliboeus* is more closely related to *Cantonius* than *Coraebus*. To confirm whether the positions of *Meliboeus* are strongly supported requires more mitogenome sequence data.

## Supporting information

S1 Fig Predicted cloverleaf structure of 22 transfer RNAs (tRNAs) in the mitogenome of *Agrilus discalis*. (TIF)

S2 Fig Predicted cloverleaf structure of 22 transfer RNAs (tRNAs) in the mitogenome of *Cantonius szechuanensis*. (TIF)

S3 Fig Predicted cloverleaf structure of 22 transfer RNAs (tRNAs) in the mitogenome of *Endelus continentalis*. (TIF)

S4 Fig Predicted cloverleaf structure of 22 transfer RNAs (tRNAs) in the mitogenome of *Habroloma* sp. (TIF)

S5 Fig Predicted cloverleaf structure of 22 transfer RNAs (tRNAs) in the mitogenome of *Sambus kanssuensis*. (TIF)

S6 Fig Phylogenetic relationships of the studied species of Buprestidae using a Maximum-likelihood (ML) method based on the PCGRNA of the mitogenomes. The numbers on the branches are the bootstrap value.
(TIF)

S7 Fig Phylogenetic relationships of the studied species of Buprestidae using the CAT-GTR model based on the cd12 + rRNAs. The values above the nodes represent the Bayesian posterior probabilities.
(TIF)

S8 Fig Morphological characteristics of adult buprestid (dorsal view). *Agrilus discalis* (A); *Endelus continentalis* (B); *Habroloma sp*. (C); *Sambus kanssuensis* (D). The scale at the lower right corner of all pictures is unified as one millimeter.
(TIF)

S1 Table Taxa used in the present study. (PDF)

S2 Table Relative synonymous codon usage (RSCU) for the protein-coding genes (PCGs) of the mitogenome of *Agrilus discalis*. (PDF)

S3 Table Relative synonymous codon usage (RSCU) for the protein-coding genes (PCGs) of the mitogenome of *Cantonius szechuanensis*. (PDF)

S4 Table Relative synonymous codon usage (RSCU) for the protein-coding genes (PCGs) of the mitogenome of *Endelus continentalis*. (PDF)

S5 Table Relative synonymous codon usage (RSCU) for the protein-coding genes (PCGs) of the mitogenome of *Habroloma* sp. (PDF)

S6 Table Relative synonymous codon usage (RSCU) for the protein-coding genes (PCGs) of the mitogenome of *Sambus kanssuensis*. (PDF)
