# Supplementary figures and images for: Mitogenomic analysis and phylogenetic relationships of Agrilinae: Insights into the evolutionary patterns of a diverse buprestid subfamily
Source: PLoS One. 2023 Sep 28;18(9):e0291820. doi: 10.1371/journal.pone.0291820 (PMC10538768; doi:10.1371/journal.pone.0291820)

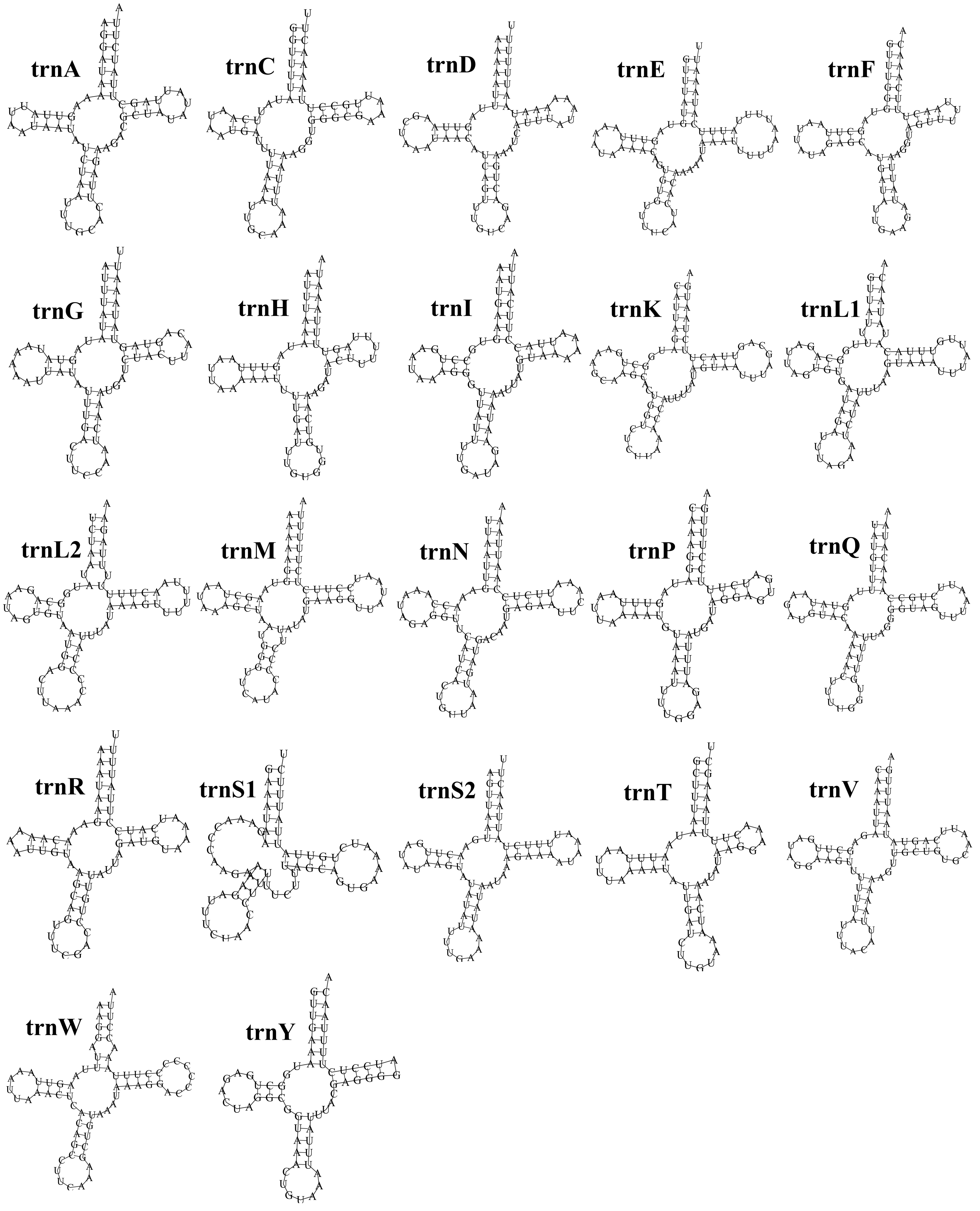

Supplement: S1 Fig — (TIF) [file pone.0291820.s001.tif]

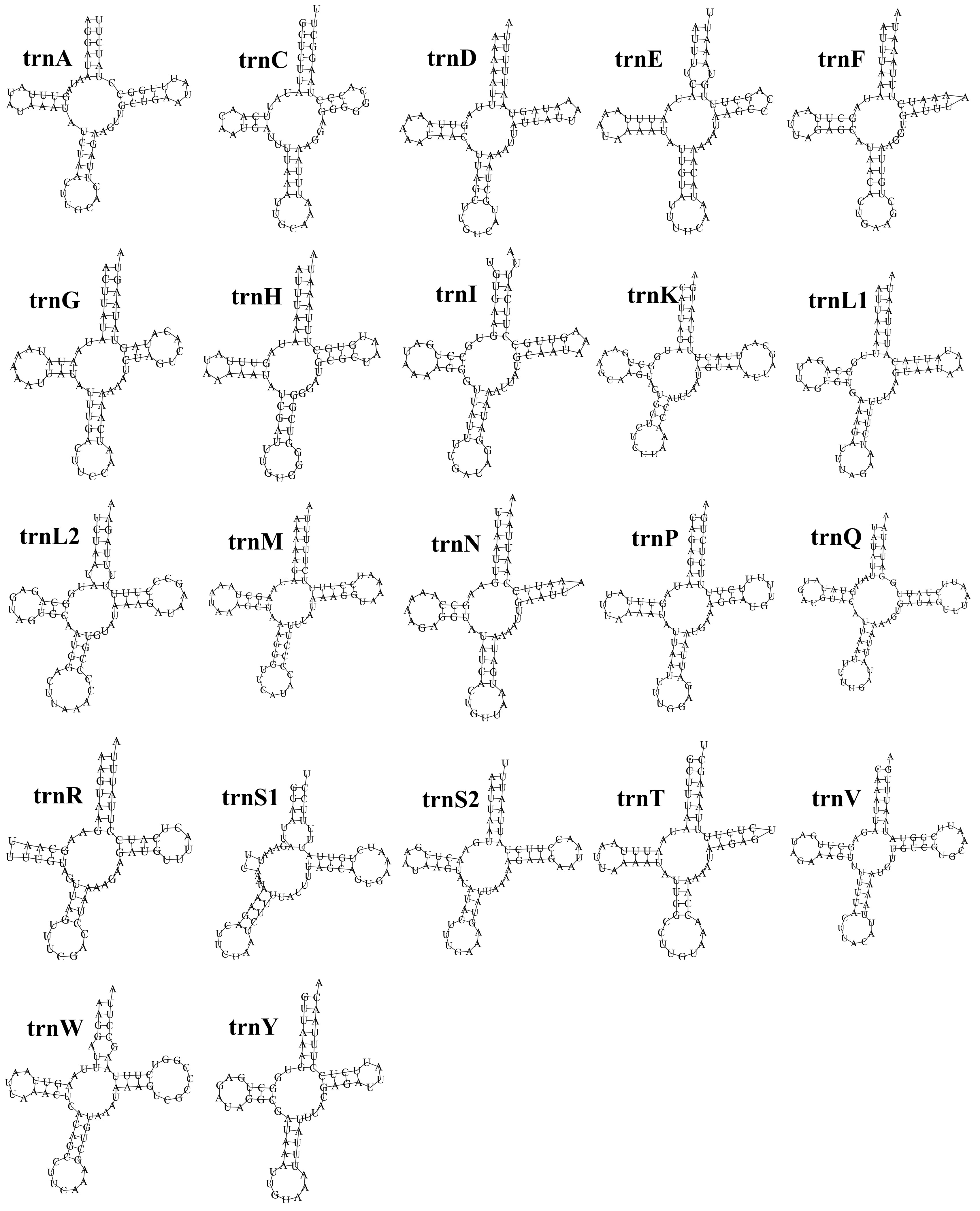

Supplement: S2 Fig — (TIF) [file pone.0291820.s002.tif]

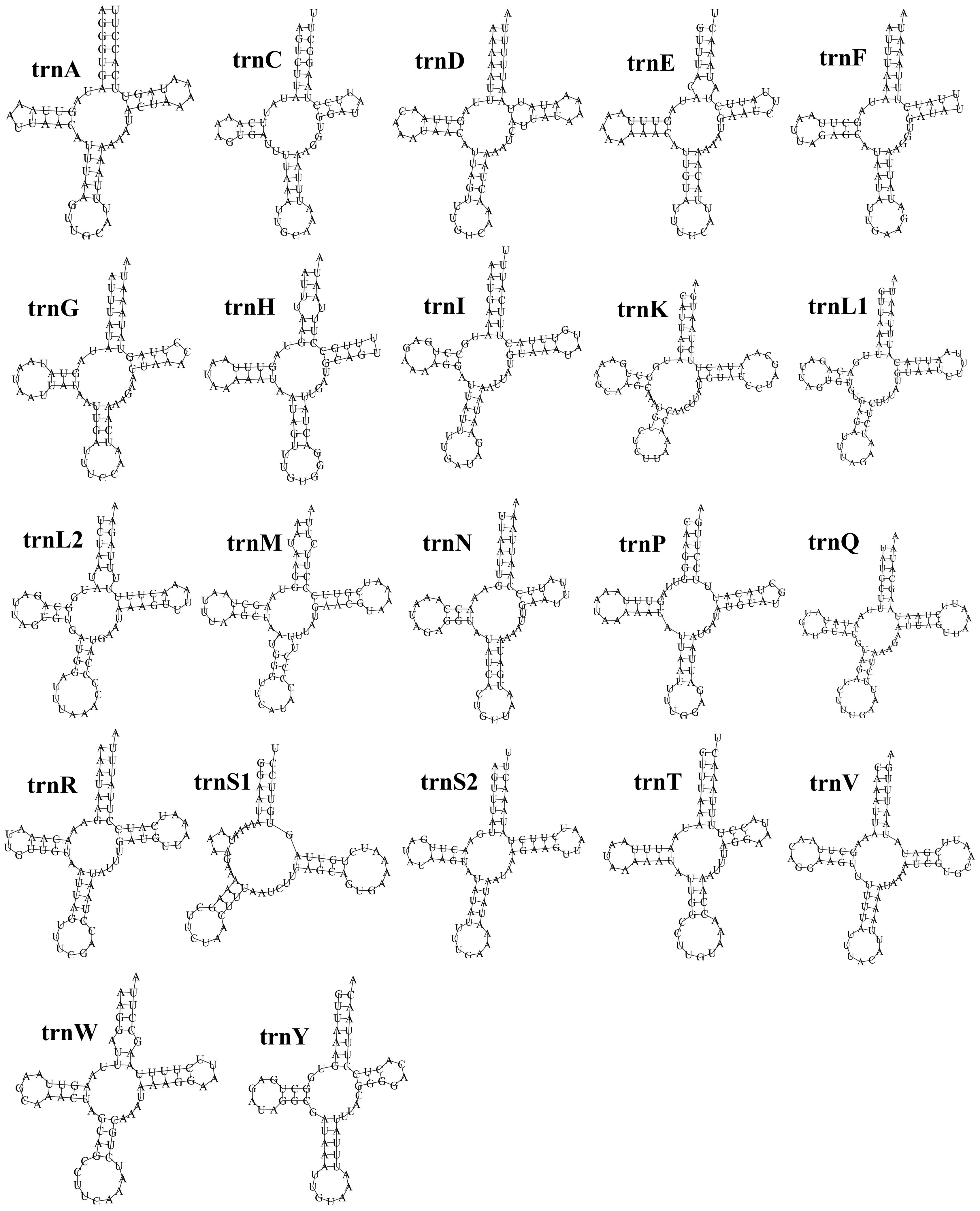

Supplement: S3 Fig — (TIF) [file pone.0291820.s003.tif]

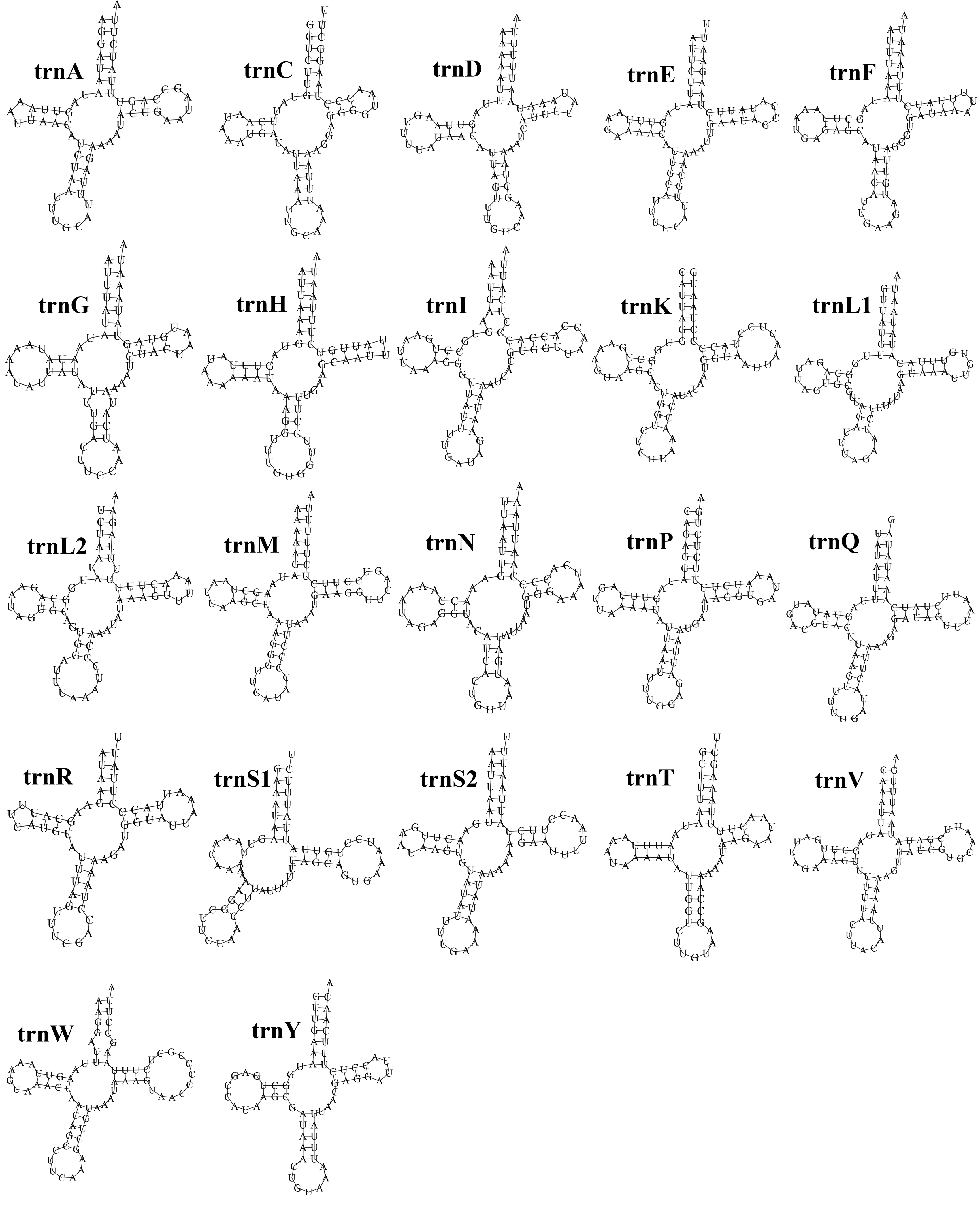

Supplement: S4 Fig — (TIF) [file pone.0291820.s004.tif]

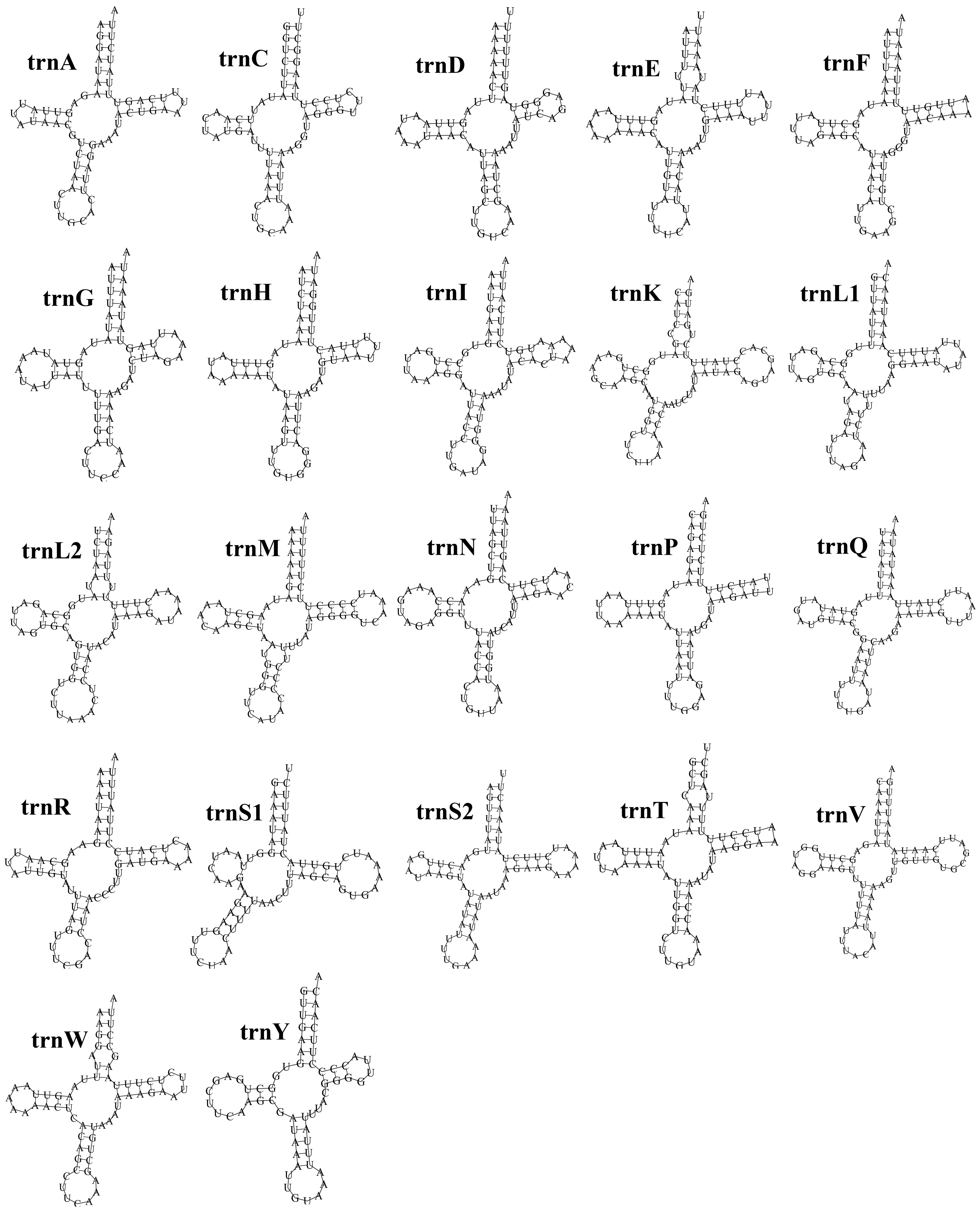

Supplement: S5 Fig — (TIF) [file pone.0291820.s005.tif]

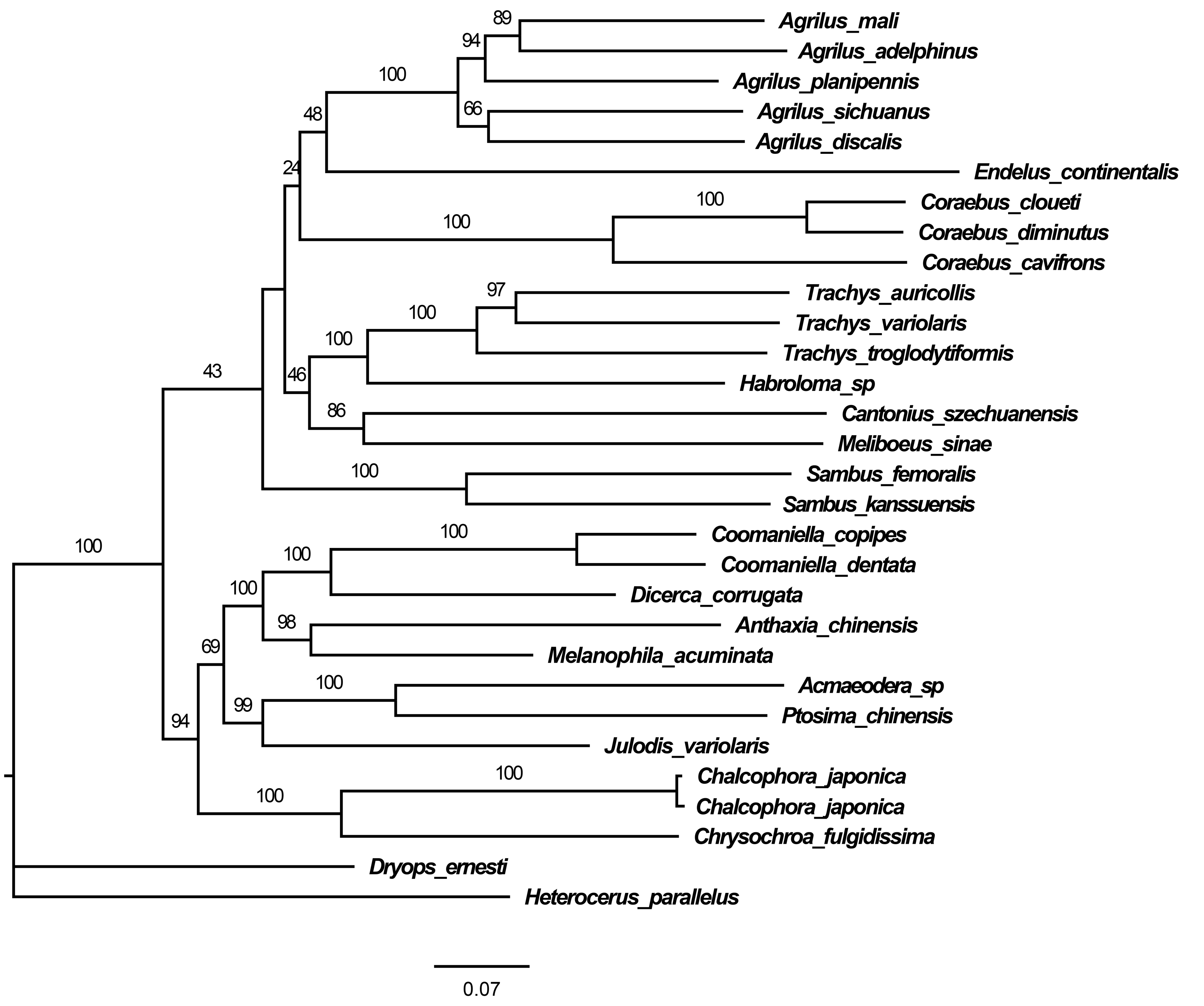

Supplement: S6 Fig — The numbers on the branches are the bootstrap value. (TIF) [file pone.0291820.s006.tif]

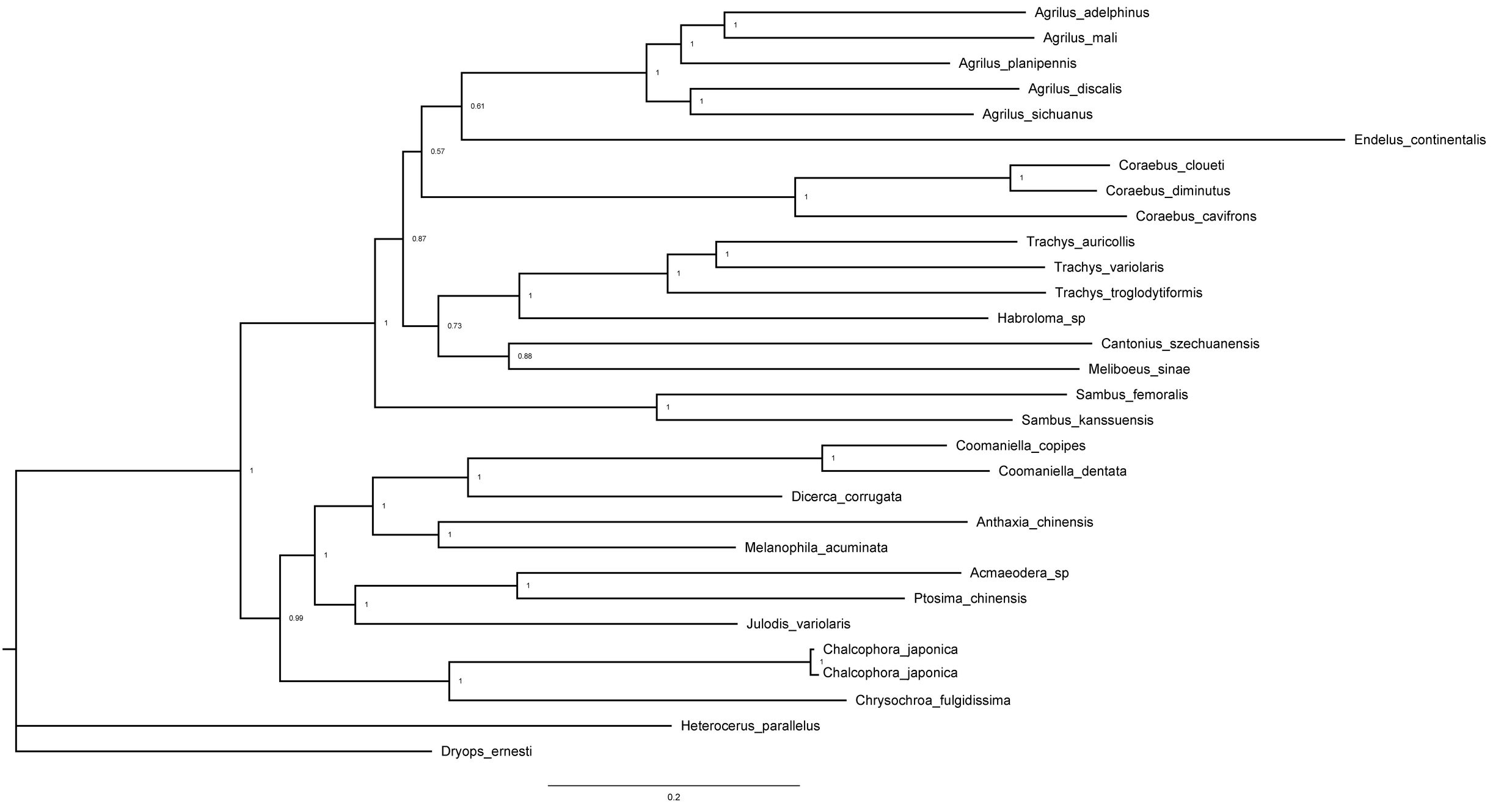

Supplement: S7 Fig — The values above the nodes represent the Bayesian posterior probabilities. (TIF) [file pone.0291820.s007.tif]

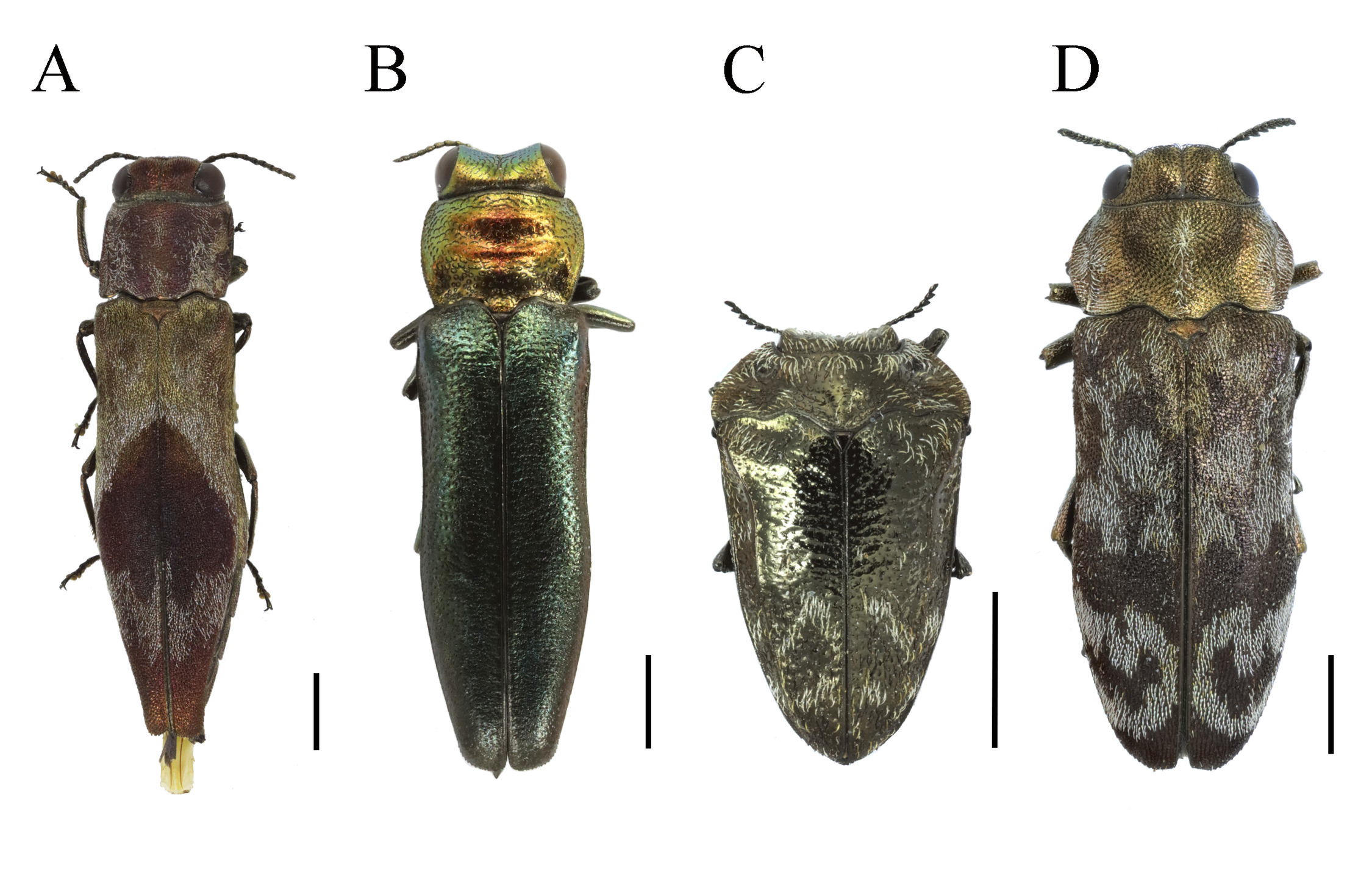

Supplement: S8 Fig — Agrilus discalis (A); Endelus continentalis (B); Habroloma sp. (C); Sambus kanssuensis (D). The scale at the lower right corner of all pictures is unified as one millimeter. (TIF) [file pone.0291820.s008.tif]
