# Supplementary material for: Mitogenomic analysis and phylogenetic relationships of Agrilinae: Insights into the evolutionary patterns of a diverse buprestid subfamily
Source: PLoS One. 2023 Sep 28;18(9):e0291820. doi: 10.1371/journal.pone.0291820 (PMC10538768; doi:10.1371/journal.pone.0291820)
Supplement: S1 Table — (PDF) [file pone.0291820.s009.pdf]

Table S1. Taxa used in the present study.

Taxa	Accession No.	Genome size (bp)	A+T%	AT-skew	References
Coraebus diminutus	OK189521	15,499	68.42	0.12	Wei 2022
Coraebus cloueti	OK189520	15,514	69.27	0.11	Wei 2022
Coraebus cavifrons	MK913589	15,686	69.79	0.12	Cao & Wang 2019a
Meliboeus sinae	OK189522	16,108	72.42	0.11	Wei 2022
Sambus femoralis	OK349489	15,367	73.23	0.12	Wei 2022
Sambus kanssuensis	OQ784265	15,411	72.40	0.10	In this study
Agrilus adelphinus	OP401219	15,732	71.35	0.10	Unpublished
Agrilus sichuanus	OK189519	16,521	71.73	0.12	Wei 2022
Agrilus planipennis	KT363854	15,942	71.90	0.12	Duan et al. 2017
Agrilus mali	MN894890	16,204	74.46	0.08	Sun et al. 2020
Agrilus discalis	ON644870	15,784	74.59	0.11	In this study
Cantonius szechuanensis	OQ784264	15,927	73.09	0.11	In this study
Endelus continentalis	OL702762	16,246	75.60	0.13	In this study
Trachys auricollis	MH638286	16,429	71.05	0.10	Xiao et al. 2019
Trachys troglodytiformis	KX087357	16,316	74.62	0.10	Unpublished
Trachys variolaris	MN178497	16,771	72.11	0.11	Cao and Wang 2019b
Habroloma sp.	OQ784266	16,273	73.99	0.11	In this study
Melanophila acuminata	MW287594	15,853	75.66	0.02	Peng et al. 2021
Anthaxia chinensis	MW929326	15,881	73.61	0.09	Chen et al. 2021
Coomaniella copipes	OL694145	16,196	74.47	0.03	Huang et al. 2022
Coomaniella dentata	OL694144	16,179	76.59	0.01	Huang et al. 2022
Chrysochroa fulgidissima	EU826485	15,592	69.92	0.15	Hong et al. 2009
Chalcophora japonica	OP388437	15,759	67.97	0.13	Wei et al. 2023
Chalcophora japonica	OM161962	15,759	67.94	0.13	Weng et al. 2022
Dicerca corrugata	OL753086	16,276	71.76	0.09	Huang et al. 2022
Acmaeodera sp.	FJ613420	16,217	68.41	0.11	Sheffield et al. 2009
Ptosima chinensis	OP388449	16,115	67.00	0.13	Wei et al. 2023
Julodis variolaris	OP390084	16,227	70.43	0.12	Wei et al. 2023
Heterocerus parallelus	KX087297	15,845	74.03	0.13	Unpublished
Dryops ernesti	KX035147	15,672	72.98	0.07	Unpublished
