# Supplementary material for: Mitogenomic analysis and phylogenetic relationships of Agrilinae: Insights into the evolutionary patterns of a diverse buprestid subfamily
Source: PLoS One. 2023 Sep 28;18(9):e0291820. doi: 10.1371/journal.pone.0291820 (PMC10538768; doi:10.1371/journal.pone.0291820)
Supplement: S4 Table — (PDF) [file pone.0291820.s012.pdf]

Table S4. Relative synonymous codon usage (RSCU) for the protein-coding genes (PCGs) of the mitogenome of *Endelus continentalis*.

Codon	Count	RSCU	Codon	Count	RSCU	Codon	Count	RSCU	Codon	Count	RSCU
UUU(F)	272	1.59	UCU(S)	95	2.07	UAU(Y)	120	1.58	UGU(C)	33	1.69
UUC(F)	71	0.41	UCC(S)	19	0.41	UAC(Y)	32	0.42	UGC(C)	6	0.31
UUA(L)	396	4.13	UCA(S)	118	2.57	UAA(*)	0	0	UGA(W)	75	1.63
UUG(L)	48	0.5	UCG(S)	2	0.04	UAG(*)	0	0	UGG(W)	17	0.37
CUU(L)	51	0.53	CCU(P)	57	1.68	CAU(H)	53	1.45	CGU(R)	15	1.2
CUC(L)	6	0.06	CCC(P)	23	0.68	CAC(H)	20	0.55	CGC(R)	3	0.24
CUA(L)	67	0.7	CCA(P)	51	1.5	CAA(Q)	57	1.87	CGA(R)	29	2.32
CUG(L)	8	0.08	CCG(P)	5	0.15	CAG(Q)	4	0.13	CGG(R)	3	0.24
AUU(I)	336	1.74	ACU(T)	71	1.44	AAU(N)	137	1.55	AGU(S)	42	0.91
AUC(I)	51	0.26	ACC(T)	30	0.61	AAC(N)	40	0.45	AGC(S)	8	0.17
AUA(M)	250	1.75	ACA(T)	92	1.87	AAA(K)	103	1.76	AGA(S)	73	1.59
AUG(M)	35	0.25	ACG(T)	4	0.08	AAG(K)	14	0.24	AGG(S)	11	0.24
GUU(V)	89	2.28	GCU(A)	65	2.05	GAU(D)	50	1.56	GGU(G)	60	1.17
GUC(V)	5	0.13	GCC(A)	15	0.47	GAC(D)	14	0.44	GGC(G)	6	0.12
GUA(V)	54	1.38	GCA(A)	44	1.39	GAA(E)	62	1.59	GGA(G)	116	2.26
GUG(V)	8	0.21	GCG(A)	3	0.09	GAG(E)	16	0.41	GGG(G)	23	0.45
